# Use of Convalescent Human Serum in Influenza Pneumonia

**Published:** 1919

**Authors:** 


**Use of Convalescent Human Serum in Influenza Pneumonia.** By Lieut. L. W. McGuire, U. S. N. and Lieut. W. R. Redden, U. S. N. *American Journal of Public Health*, October 1918.

The authors treated in all over 400 cases of influenza pneumonia at the hospital; the mortality in the earlier cases was as high as 50 to 60 o/o, later dropping to 30 o/o.

In the serum treatment, " convalescent patients were bled as soon as convalescence was established, the majority within a week or ten days of a drop to normal temperature ". The serum was given as early as the diagnosis of the pneumonia complication could be made. The dose of serum varied from 75 cc. to 125 cc. intravenously; interval between doses from eight to sixteen hours; treatment continued " until there was no doubt about the recovery of the patient ". Majority received 300 cc. in all; two from 600 to 700 cc.; three only 100 cc. If no results are obtained from serum in 24 hours, serum of another donor should be used.

All donors received Wassermann test; those with positive reaction not used. Compatibility tests of the sera of several donors were made with each recipient's corpuscles as soon as new cases arrived in the ward.

Up to the time of the report thirty-seven pneumonia cases had been treated; thirty had recovered; one died; six were under treatment, all but one progressing favorably.

The authors conclude that : —

" At present the potency of the convalescent serum can be tested only by its clinical effect.

" Most beneficial results will be obtained by giving the proper serum within the first 48 hours of the pneumonia complication. The virulence of the organism apparently decreased as the epidemic progressed, but, making allowance for this decreasing virulence, it is believed that the serum from convalescent influenza pneumonia patients has a decided influence in shortening the course of the disease and in lowering mortality. "

This treatment requires the co-operation of a well equipped laboratory.

